# The Anticancer Role of Cerium Oxide Nanoparticles by Inducing Antioxidant Activity in Esophageal Cancer and Cancer Stem-Like ESCC Spheres

**DOI:** 10.1155/2022/3268197

**Published:** 2022-12-01

**Authors:** Hossein Javid, Seyed Isaac Hashemy, Mohammad Foad Heidari, Ali Esparham, Sattar Gorgani-Firuzjaee

**Affiliations:** ^1^Department of Clinical Biochemistry, Faculty of Medicine, Mashhad University of Medical Sciences, Mashhad, Iran; ^2^Medical Laboratory Sciences Department, Varastegan Institute for Medical Sciences, Mashhad, Iran; ^3^Surgical Oncology Research Center, Mashhad University of Medical Sciences, Mashhad, Iran; ^4^Medical Laboratory Sciences, School of Allied Health Medicine, AJA University of Medical Sciences, Tehran, Iran; ^5^DNA Molecular Identification Center, Aja University of Medical Sciences, Tehran, Iran; ^6^Student Research Committee, Faculty of Medicine, Mashhad University of Medical Sciences, Mashhad, Iran; ^7^Clinical Biochemistry, School of Allied Medical Sciences, Infectious Diseases Research Center, AJA University of Medical Sciences, Tehran, Iran; ^8^Infectious diseases research Center, AJA university of Medical Sciences, Tehran, Iran

## Abstract

**Introduction:**

Esophagus squamous cell carcinoma (ESCC) has a poor prognosis, a high rate of metastasis, and rapid clinical progression. One hypothesis is that therapeutic failure is due to the presence of cancer stem cells (CSC). Previous studies showed the anticancer effect of cerium oxide nanoparticles (CNP) in different cancer cells. In this study, we aim to evaluate the effect of cerium oxide nanoparticles on cell antioxidants, toxicity, as well as cell oxidant level in esophageal cancer (YM1) and cancer stem cell-like (CSC-LC) cell lines.

**Method:**

YM1 and CSC-LC spheres were treated with CNP at different concentrations. The cell viability was assessed by using the MTT test. Antioxidant levels (SOD (superoxide dismutase, CAT (catalase), thiol, and TAC (total antioxidant capacity)), antioxidant genes expression (SOD and CAT), ROS (reactive oxygen species), and MDA (malondialdehyde) levels were assessed in both cell lines.

**Results:**

CSC-LC had significantly elevated SOX4 and OCT4 pluripotent genes. The ROS and MDA levels were significantly reduced in both YM1 and CSC-LC spheres after treatment with CNP. Also, the antioxidant levels and expressions were elevated significantly in both cell lines after CNP treatment.

**Conclusion:**

These results suggest the potential anticancer effect of CNP by elevating antioxidant levels and expressions, and reducing oxidant levels.

## 1. Introduction

Esophageal cancer (EC) is the eighth most common cancer and the sixth most common cause of cancer-related death worldwide [1]. Esophagus squamous cell carcinoma (ESCC), the most frequent type of EC, has a poor prognosis, a high rate of metastasis, and rapid clinical progression [2]. The global incidence of ESCC was reported to be 87% of all EC cases in 2012 [3]. Despite the progression of early detection, surgery, and chemotherapy in patients with ESCC, its prognosis remains poor and challenging [4]. One hypothesis is that therapeutic failure is due to the presence of cancer stem cells which can cause recurrence, distant metastasis, and therapy resistance [5]. These cells have the ability to maintain and induce malignancy proliferation and metastasis in different types of cancers [6]. Finding a novel treatment to eliminate these cancer stem cells can help find new diagnostic and treatment approaches [7]. Nanotechnology has become a main focus of biomedical research area in recent years and its applications include drug delivery systems, tissue engineering, and luminescent biomarkers, among others [8].

The free radicals play a critical role in killing bacteria and viruses, as well as activation of enzymes and hormones, and producing energy [9]. They also have an important role in cell homeostasis and cell signal transductions [10]. The levels of these free radicals and reactive oxygen species (ROS) are controlled by antioxidant agents in human cells. An imbalance between ROS and the antioxidant agent is defined as oxidative stress, which has been linked to cardiovascular disease, neurodegenerative disease, diabetes mellitus, and different types of cancers [11, 12]. It was shown that cancer cells have elevated levels of ROS in comparison to normal nontransformed cells [13]. In addition to the impact of ROS on the genome, it can play a role in promoting cancer cell proliferation, angiogenesis, survival, and metastasis [14]. However, ROS can have a contrary effect on cancer cells. Excessive levels of ROS can induce cancer cell death by increasing cell oxidative stress [15]. To prevent cancer cell death, they increase the level of antioxidant capacity to scavenge excessive ROS. Therefore, cancer cells have elevated levels of both ROS and antioxidants and this feature can make cancer cells more sensitive to ROS levels alteration [16, 17].

Cerium, as a lanthanide rare earth metal, has two oxide forms with crystalline fluorite lattice structure. In particular, cerium oxide nanoparticles (CNP) consist of a cerium core surrounded by an oxygen lattice [18]. It has been shown that CNP has several antioxidant roles including catalase mimetic activity, superoxide dismutase (SOD) activity, hydroxyl radical scavenging, and nitric oxide radical scavenging. However, other studies revealed that CNP has a cytotoxicity role for cancer cells, an antiinvasive role, sensitizing role to radiation for cancer cells, in addition to protecting other surrounding normal cells [19].

In this study, we aim to evaluate the effect of cerium oxide nanoparticles on cell antioxidant (SOD, TAC (total antioxidant capacity), thiol, and CAT (catalase)), toxicity, as well as cell oxidant (ROS and MDA (malondialdehyde)) levels in esophageal cancer (YM1) and cancer stem cell-like (CSC-LC) cell lines.

## 2. Methods and Materials

### 2.1. Cell Culture and Cerium Oxide Nanoparticles

The present study was conducted at Mashhad University of Medical Science, Mashhad, Iran. The YM1 human esophageal squamous cell carcinoma cell line was previously established in our lab at Golestan University of Medical Sciences [20]. A 1 : 1 mixture of RPMI 1640, and Ham's F-12 (Betacell, Iran) was supplemented with 1% penicillin-streptomycin (Grand Island, NY, USA) and 5% fetal bovine serum for 5 to 6 days until the exponential growth phase (0.6–1 × 106 cells/mL). The synthesized CNPs were kindly received from Dr. Darroudi and characterized by Fourier transform infrared (FT-IR) (Tensor27, Bruker, Germany), powder X-ray diffraction (PXRD) (The Netherlands, PANalyticalX'Pert PRO MPD system, Cu K*α*), Raman (Takram P50C0R10, laser wavelength = 532 nm), and field emission scanning electron microscopy (FESEM; MIRA3 TESCAN, Czech Republic) [21, 22]. The biological experiments were conducted by using a microplate reader (BioChrom Anthos 2020 MicroPlate Reader, UK), and an invert microscope (HUND, Germany).

### 2.2. ESCC Cell Sphere Formation

The polymer of poly HEMA (2-Hydroxyethyl methacrylate) coated Petri dishes were used for transferring single cell suspensions derived from adherent cells with a concentration of 100000 cells/mL for ESCC cells. The cells were maintained in the following solution at 37°C with 5% CO_2_ to form spheres: serum-free RPMI/F12 medium supplemented with 20 ng/mL basic fibroblast growth factor (Grand Island, NY, USA), 2% B-27 supplement (Grand Island, NY, USA), and 20 ng/mL epidermal growth factor (Sigma-Aldrich Company, USA). Every two days, the medium was refreshed to replenish nutrients. Following the separation of the spheres into single cells, after 6 days, they were cultured in the new nonadherent Petri dishes with the same before condition. The stem-like properties of sphere cells were ready for the following experiments after three passages.

### 2.3. Cell Viability Assay

Resazurin assay was used to investigate the cell toxicity of CNPs. Briefly, 25000 cells/well were seeded in a 96-well plate. After 24 hours of incubation at 37°C, CNPs were inoculated into the grown cells with different concentrations (0, 15.6, 31.2, 62.5, 125, and 250 *μ*g/mL). Then, each well received the resazurin solution (phosphate buffer saline, 0.01 mg/mL) every 24 and 48 hours of incubation. The medium was discarded after finishing the incubation. Afterward, the resazurin solution (phosphate buffer saline, 0.01 mg/mL) was added to each well. After shaking the plates for 3 minutes, the optimal absorbance in the subsequent 3 hours was recorded at 600 nm excitation and 570 nm emission on a Perkin Elmer fluorimeter, and the IC_50_ value was evaluated by using the GraphPad Prism® 6 (GraphPad Software, San Diego, CA, USA) software.

### 2.4. ROS Level Assay

Intracellular ROS production level was measured by using a 2′,7′-Dichlorodihydrofluorescein diacetate (DCFDA) cellular ROS detection assay based on the manufacturer's protocol. Accordingly, 20 *μ*M DCFDA was exposed to cells and then DCFDA was washed after 24 hours of incubation. Afterward, the CNPs (100 *μ*M, 300 *μ*M, 500 *μ*M, 700 *μ*M, and 1000 *μ*M) were added to rewashed cells for 24 hours. Also, the positive control consists of tert-butyl hydroperoxide (TBHP). Finally, by using the fluorescence plate reader Perkin Elmer, the relative fluorescence intensity was recorded for these groups.

### 2.5. MDA Level Assay

The commercial kit r (Teb Pazhouhan Razi, Tehran, Iran) was used to assess the MDA level as a marker of oxidative stress level based on the manufacturer's protocol. Briefly, the cells were treated with the CNPs (100 *μ*M, 300 *μ*M, 500 *μ*M, 700 *μ*M, and 1000 *μ*M) and after 24 hours of incubation, 1× Butylated hydroxytoluene (BHT) was added to lyse the cells. Subsequently, after mixing with 500 *μ*L TCA, the prepared sample was incubated at 95°C for 5 min. The mixture was centrifuged (14000 g) for 5 min. After adding thiobarbituric acid (TBA) to the supernatants, the mixture was incubated at 95°C for 30 min. Finally, a spectrophotometer was used to measure the absorption of mixtures at 532 nm. The MDA level was measured by using a standard curve from GraphPad Prism software.

### 2.6. Antioxidant Gene Expression Assay

The SOD and CAT gene expressions were analyzed in both YM1 and CLC-SC cells which were treated with CNPs. After culturing the cells in a 6-well plate in RMPI medium at 65 × 10^4^ cells/well, CNP was incubated at different concentrations (0, 100, 500, 700, and 1000 *μ*g/mL) for 24 hours. The cells were washed twice with phosphate-buffered saline (PBS, 0.1 M, pH 7.2). RNA was extracted from the treated cells with the manufacturer's instructions (Parstous, Mashhad, Iran). Afterward, a real-time polymerase chain reaction (PCR) was done for samples as described in the previous study [23, 24].

### 2.7. Antioxidant Activity Assay (SOD, CAT, TAC, and Thiol)

#### 2.7.1. SOD Activity Assay

The SOD assay kit (Teb Pazhouhan Razi, Tehran, Iran) was utilized for measuring SOD activity after treating the cells with CNPs based on the instructor's protocol. By using a plate reader, the absorbance of the samples was measured at 450 nm.

#### 2.7.2. CAT Activity Assay

The CAT assay commercial kit (Teb Pazhouhan Razi, Tehran, Iran) was used to assess the CAT enzyme activity following the manufacturer's protocol. Teb Pazhouhan Razi kit works based on hydrogen peroxide (H_2_O_2_) decomposition. The samples' absorbance was assessed at 450 nm.

#### 2.7.3. TAC Activity Assay

By using TAC commercial kit assay (Teb Pazhouhan Razi, Tehran, Iran), the level of TAC was measured. Briefly, after the treatment of cell lines with CNPs, cells were lysed by freezing and thawing. The samples were centrifuged (12000 g) for 15 min. Afterwards, according to the manufacturer's instructions, the required reagents were added to supernatants. Finally, the absorbance of samples was measured and the level of TAC was estimated by a standard curve.

#### 2.7.4. Thiol Activity Assay

Thiol groups' total level was estimated by the DTNB (2,2′-dinitro-5,5′-dithiol-benzoic acid) (Teb Pazhouhan Razi, Tehran, Iran) reduction method. The reaction of DTNB with SH groups results in yellow color. Initially, 0.1 mL of Tris–EDTA buffer (pH 8.6) and 0.05 mL prepared cell lysate were mixed and the samples' absorbance was measured against Tris–EDTA buffer (A1) at 412 nm. Afterward, 20 *μ*L DTNB was added to the mixture and the mixture was incubated for 15 minutes at room temperature. Remeasurement was done for all the samples. Of note, blank group (B) consisted of the absorbance of the DTNB reagent. The following formula was used to assess total SH levels(*μ*M):

(1) $$\begin{array}{c} \text{Total thiol concentration }\left(\mu \text{M}\right)=\left(\text{A}2‐\text{A}1‐\text{B}\right)\times 1.07/0.05\times 13.6. \end{array}$$

### 2.8. Statistical Analysis

The experimental data are shown as mean ± standard error of the mean. The data were analyzed by ANOVA test followed by Bonferroni's *t*-test and the GraphPad Prism® 6.0 software (San Diego, CA, USA) for Windows. All the results were analyzed triplicate in comparison to the untreated control group. A *p* value lower than 0.05 was used for a statistically significant level.

## 3. Results

### 3.1. Tumor Spheres Showed CSC-LC Features

**Figure 1:**
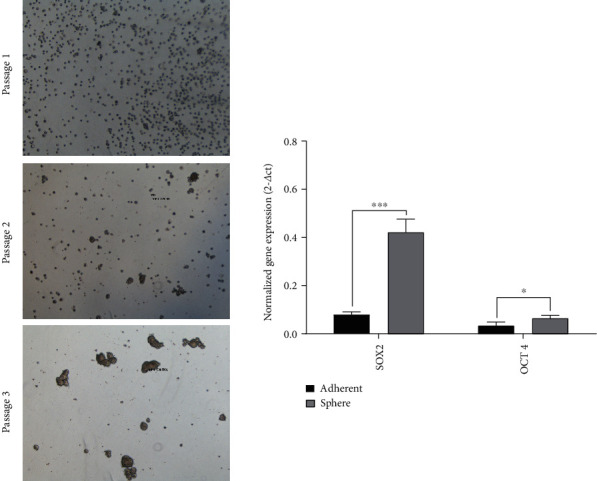
CSC characterization of YM1 cells. Section (a): the enrichment process of CSCs is shown. The sphere was developed from passages 1 to 3. Section (b): the pluripotency regulators (SOX2 and OCT4) were significantly overexpressed in sphere cells in comparison to the attached cells. (^∗^*p* value < 0.05, ^∗∗^*p* value < 0.01, ^∗∗∗^*p* value< 0.001, ^∗∗∗∗^*p* value < 0.0001) (CSC: cancer stem cell).

Firstly, the corresponding adherent cells were compared with the YM1 derived sphere in passage three in order to confirm the CSCs enrichment. As indicated in Figure 1, prominent well-shaped spheres can be seen after three passages. The level of SOX2 and OCT4 have assessed in both passage 3 spheres and the corresponding adherent cells to characterize the stemness of spheres and the qRT-PCR demonstrated that both SOX2 and OCT4 were significantly overexpressed in sphere cells. The upregulation of pluripotency genes characterized the sphere cells and CSC-LC was confirmed by anchorage independent growth characteristics in consequent experiments.

### 3.2. CSC-LC and YM1 Cell Lines Viability Assay

**Figure 2:**
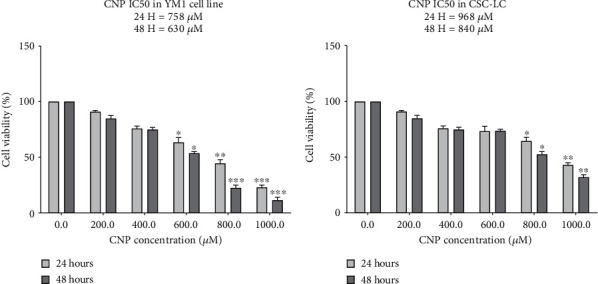
The resazurin assay result shows the effect of CNPs on cell viability of YM1 and CSC-LC cell lines. CNPs can cause ESCC cell death in both YM1 (ESCC) and CSC-LC cell lines in a dose- and time-dependent manner. Section (a): IC_50_s in CSC-LC treated with CNPs are 968 *μ*M and 840 *μ*M after 24 and 48 hours, respectively. Section (b): IC_50_s in YM1 cell line treated with CNPs are 758 *μ*M and 630 *μ*M after 24 and 48 hours, respectively. (^∗^ shows a significant difference between each concentration and the control group) (^∗^*p* value < 0.05, ^∗∗^*p* value < 0.01, ^∗∗∗^*p* value < 0.001, ^∗∗∗∗^*p* value < 0.0001) (CNP: cerium oxide nanoparticle).

Figure 2 summarized the results of resazurin assays for CSC-LC and YM1 cell lines with different concentrations of CNP (0, 200, 400, 600, 800, and 1000 *μ*M) after 24 and 48 hours of cell inoculation. The results showed that CNP at concentrations of 800 and 1000 *μ*M could decrease the viable CSC-LCs significantly. Also, the viable YM1 cells decreased significantly with 600, 800, and 1000 *μ*M of CNP. IC_50_s for CSC-LC were 968 and 840 *μ*M after 24 and 48 hours, respectively (Figure 2(a)). Also, for the YM1 cell line, the IC_50_s were 758 and 630 *μ*M after 24 and 48 hours, respectively (Figure 2(b)).

### 3.3. The ROS Levels in CSC-LC and YM1 Cell Lines

**Figure 3:**
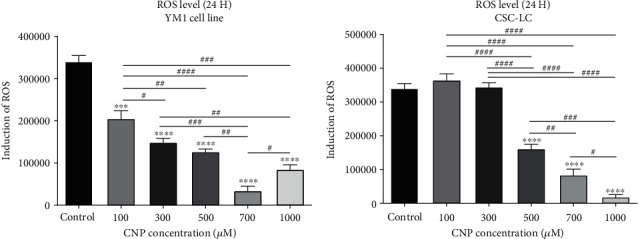
The effect of CNPs on ROS level in YM1 and CSC-LC cell lines. ROS level decreased significantly in both cell lines in comparison to the control (untreated cells). Section (a): the level of intracellular ROS in YM1 cell line. Section (b): the level of intracellular ROS in CSC-LC. (^∗^ shows a significant difference between each concentration and the control group and ^#^ shows a significant difference between different concentrations) (^∗^ or ^#^*p* value < 0.05, ^∗∗^ or *^##^* *p* value < 0.01, ^∗∗∗^ or ^###^*p* value < 0.001, ^∗∗∗∗^ or ^####^*p* value < 0.0001) (CNP: cerium oxide nanoparticle, ROS: reactive oxygen species).

The ROS level was assessed in both CSC-LC and YM1 cell lines to elucidate the effect of CNP on cell oxidative products with H_2_DCFDA staining. Our results showed that CNP at concentrations equal to or above 100 *μ*M was significantly associated with decreased ROS levels in YM1 cell lines, as shown in Figure 3(a). Whereas, in CSC-LC spheres, significantly decreased ROS levels were observed at concentrations higher than 500 *μ*M of CNP (Figure 3(b)).

### 3.4. CAT Expression and Activity in CSC-LC and YM1 Cell Lines

**Figure 4:**
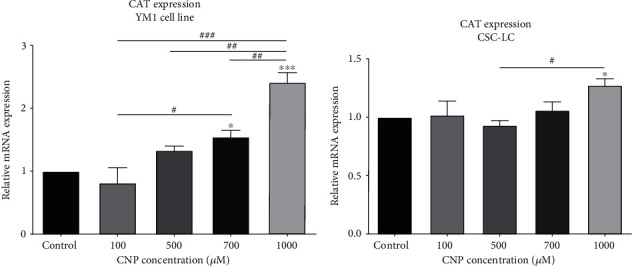
The effect of CNPs on CAT expression in YM1 and CSC-LC cell lines. The results show the upregulation of CAT expression after treatment with CNPs in both YM1 and CSC-LC cell lines in comparison to the control (untreated cells). Section (a): the CAT expression in YM1 cell line. Section (b): the CAT expression in CSC-LC. (^∗^ shows a significant difference between each concentration and the control group and ^#^ shows a significant difference between different concentrations) (^∗^ or ^#^*p* value < 0.05, ^∗∗^ or ^##^*p* value < 0.01, ^∗∗∗^ or ^###^*p* value < 0.001, ^∗∗∗∗^ or ^####^*p* value < 0.0001) (CNP: cerium oxide nanoparticle, CAT: catalase).

**Figure 5:**
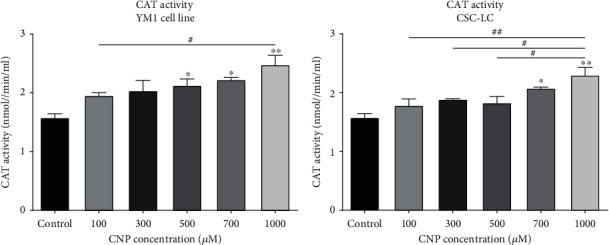
The effect of CNPs on CAT activity in YM1 and CSC-LC cell lines. CAT activity increased significantly in both YM1 and CSC-LC cell lines in comparison to the control (untreated cells). Section (a): CAT activity in YM1 cell line. Section (b): CAT activity in CSC-LC. (^∗^ shows a significant difference between each concentration and the control group and ^#^ shows a significant difference between different concentrations) (^∗^ or ^#^*p* value < 0.05, ^∗∗^ or ^##^*p* value < 0.01, ^∗∗∗^ or ^###^*p* value < 0.001, ^∗∗∗∗^ or ^####^*p* value < 0.0001) (CNP: cerium oxide nanoparticle, CAT: catalase).

Figures 4 and 5 summarized the results of CAT expression and activity in CSC-LC and YM1 cell lines, respectively. The qRT-PCR showed that CAT expression was significantly higher at concentrations of 700 and 1000 *μ*M of CNP in YM1 cell lines (Figure 4(a)). Also, in CSC-LC treated with CNP with 1000 concentration was followed by significantly higher CAT expression (Figure 4(b)). Also, identical results were seen in assessing the CAT activity in CSC-LC and YM1 cell lines (Figures 5(a) and 5(b)).

### 3.5. SOD Expression and Activity in CSC-LC and YM1 Cell Lines

**Figure 6:**
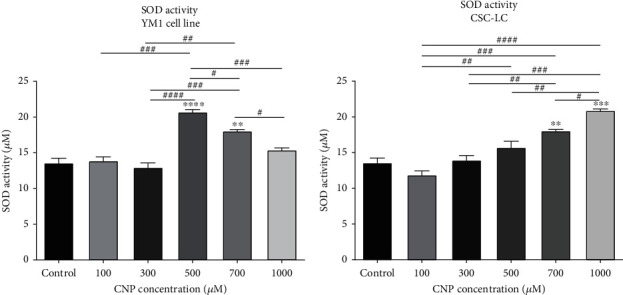
The effect of CNPs on SOD activity in YM1 and CSC-LC cell lines. SOD activity increased significantly after treatment with CNPs in both cell lines in comparison to the control (untreated cells). Section (a): SOD activity in YM1 cell line. Section (b): SOD activity in CSC-LC. (^∗^ shows a significant difference between each concentration and the control group and ^#^ shows a significant difference between different concentrations) (^∗^ or ^#^*p* value < 0.05, ^∗∗^ or ^##^*p* value < 0.01, ^∗∗∗^ or ^###^*p* value < 0.001, ^∗∗∗∗^ or ^####^*p* value < 0.0001) (CNP: cerium oxide nanoparticle, SOD: superoxide dismutase).

**Figure 7:**
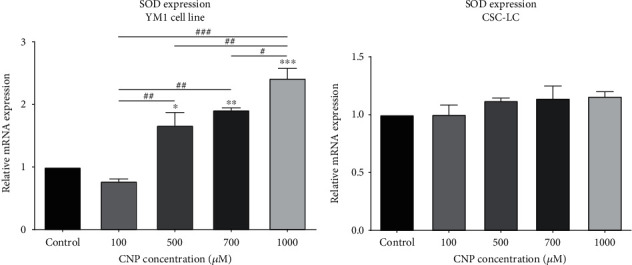
The effect of CNPs on SOD expression in YM1 and CSC-LC cell lines. Contrary to CSC-LC, SOD expression increased significantly after treatment with CNPs in YM1 cell line in comparison to the control (untreated cells). Section (a): SOD expression in YM1 cell line. Section (b): SOD expression in CSC-LC. (^∗^ shows a significant difference between each concentration and the control group and ^#^ shows a significant difference between different concentrations) (^∗^ or ^#^*p* value < 0.05, ^∗∗^ or ^##^*p* value < 0.01, ^∗∗∗^ or ^###^*p* value < 0.001, ^∗∗∗∗^ or ^####^*p* value < 0.0001) (CNP: cerium oxide nanoparticle, SOD: superoxide dismutase).

As demonstrated in Figure 6(a), SOD activity was significantly higher in YM1 cell lines treated with CNP at concentrations of 500 and 700 *μ*M. Moreover, the CSC-LC spheres which were treated with CNP at concentrations of 700 and 1000 *μ*M, have significantly higher SOD activity (Figure 6(b)). Also, SOD expression was measured in CSC-LC and YM1 cell lines with qRT-PCR which showed SOD was significantly overexpressed in YM1 cell lines treated with 500, 700, and 1000 *μ*M of CNP (Figure 7(a)). However, in CSC-LC spheres, SOD expression differences were insignificant between different concentrations of CNP (Figure 7(b)).

### 3.6. MDA Activity in CSC-LC and YM1 Cell Lines

**Figure 8:**
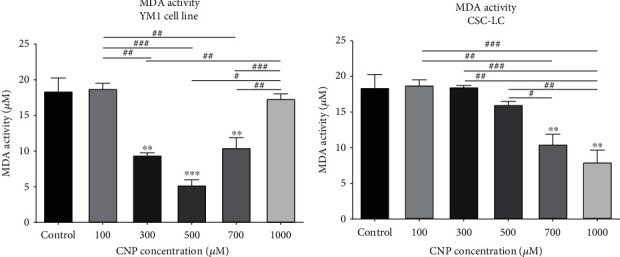
The effect of CNPs on MDA activity in YM1 and CSC-LC cell lines. The result shows MDA activity decreased significantly after treatment with CNPs in both cell lines in comparison to the control (untreated cells). Section (a): MDA activity in YM1 cell line. Section (b): MDA activity in CSC-LC. (^∗^ shows a significant difference between each concentration and the control group and ^#^ shows a significant difference between different concentrations) (^∗^ or ^#^*p* value < 0.05, ^∗∗^ or ^##^*p* value < 0.01, ^∗∗∗^ or ^###^*p* value < 0.001, ^∗∗∗∗^ or ^####^*p* value < 0.0001) (CNP: cerium oxide nanoparticle, MDA:malondialdehyde).

As shown in Figure 8(a), the YM1 cells which were treated with CNP at concentrations of 300, 500, and 700 *μ*M had significantly lower MDA activity. Also, MDA activity was significantly reduced in CSC-LCs treated with CNP at concentrations of 700 and 1000 *μ*M of CNP (Figure 8(b)).

### 3.7. TAC and Thiol in CSC-LC and YM1 Cell Lines

**Figure 9:**
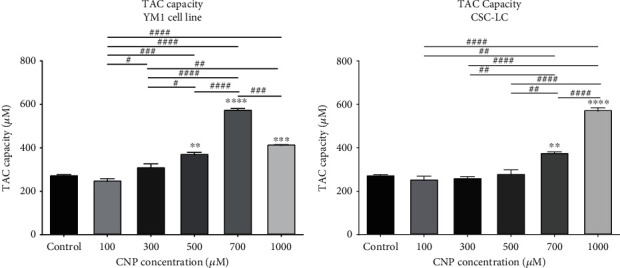
The effect of CNPs on TAC capacity in YM1 and CSC-LC cell lines. The result shows TAC capacity increased significantly after treatment with CNPs in both cell lines in comparison to the control (untreated cells). Section (a): TAC capacity in YM1 cell line. Section (b): TAC capacity in CSC-LC. (^∗^ shows a significant difference between each concentration and the control group and ^#^ shows a significant difference between different concentrations) (^∗^ or ^#^*p* value < 0.05, ^∗∗^ or ^##^*p* value < 0.01, ^∗∗∗^ or ^###^*p* value < 0.001, ^∗∗∗∗^ or ^####^*p* value < 0.0001) (CNP: cerium oxide nanoparticle, TAC: total antioxidant capacity).

**Figure 10:**
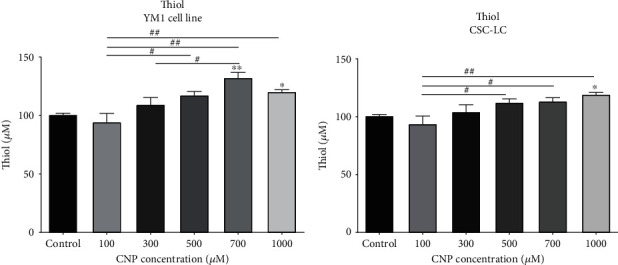
The effect of CNPs on thiol in YM1 and CSC-LC cell lines. The result shows thiol increased significantly after treatment with CNPs in both cell lines in comparison to the control (untreated cells). Section (a): thiol in YM1 cell line. Section (b): thiol in CSC-LC. (^∗^ shows a significant difference between each concentration and the control group and ^#^ shows a significant difference between different concentrations) (^∗^ or ^#^*p* value < 0.05, ^∗∗^ or ^##^*p* value < 0.01, ^∗∗∗^ or ^###^*p* value < 0.001, ^∗∗∗∗^ or ^####^*p* value < 0.0001) (CNP: cerium oxide nanoparticle).

As shown in Figure 9(a), the TAC capacity level was significantly elevated in YM1 cells treated with 500, 700, and 1000 *μ*M of CNP. Moreover, CSC-LCs treated with CNP at concentrations of 700 and 1000 *μ*M had significantly higher levels of TAC capacity (Figure 9(b)). Furthermore, measuring thiol levels revealed that YM1 cells that were treated with 700 and 1000 *μ*M of CNP, have significantly elevated levels of thiol, as shown in Figure 10(a). Also, in CSC-LC spheres, thiol increased significantly at 1000 *μ*M of CNP (Figure 10(b)).

## 4. Discussion

As previously noted EC is the sixth leading cause of cancer death worldwide and cancer stem cells are a subpopulation of cancer cells that are responsible for metastasis and treatment resistance [1, 5]. Surgery, chemotherapy, and radiotherapy are usually used for cancer treatment but they have limited efficacy and side effects and may damage normal tissues [25]. Recent studies showed the significant antitumoral effect of CNP nanoparticles in several cancer cell lines [26–29]. Contrary to conventional cancer treatments, CNPs did not have a toxic effect on healthy cells [30]. To the best of our knowledge, this is the first study that is aimed at evaluating the antitumoral effect of CNPs in YM1 (ESCC) and ESCC cell spheres (CSC-LC).

In this study, the resazurin cytotoxicity assay showed CNP could cause ESCC cell death in both YM1 (ESCC) and CSC-LC cell lines in a dose- and time-dependent manner. The ROS and MDA levels significantly decreased in both YM1 cell line and CSC-LC spheres after incubation with CNP. Also, the level of SOD, CAT, thiol, and TAC significantly increased in both YM1 and CSC-LC spheres after treatment with CNP. Further examinations revealed the gene expressions of SOD and CAT were significantly elevated in cancer cells treated with CNP. However, in CSC-LC spheres, SOD expression did not change significantly after CNP incubation. An explanation for this result is that posttranscriptional modifications such as glycation, sulfation, and phosphorylation can alter the protein behavior. Therefore, as a result of posttranscriptional modifications, some gene expression changes cannot be detected by RNA analysis [23, 31].

ROS can initiate and progress cancer cell growth, as well as downregulate the antioxidant enzymes [32]. The healthy cells intensely control the level of ROS by using antioxidants including SOD, CAT, thiols, glutathione, and peroxidase [10]. It has been indicated that cancer cells including ESCC have elevated levels of ROS in comparison to healthy cells which may be the result of mitochondria dysfunction, increased metabolic activities, elevated peroxisome activity, increased cell signaling, and oncogenes activity [33]. Furthermore, ROS can induce genetic instability, proliferation, angiogenesis, and metastasis in cancer cells [34]. With regard to the double-edged sword character of ROS in the treatment of cancer cells, which will be explained later, lowering and elevating strategies have been suggested for cancer treatment.

Previous studies showed the antiapoptotic effect of high levels of ROS which is the result of redox-sensitive transcription activation including nuclear factor *κ*-light-chain-enhancer of activated B cells (NF-*κ*B) [35]. NF-*κ*B as an important transcription factor can inhibit apoptosis by regulating antiapoptotic genes such as *Bcl-2* and *survivin* [36, 37]. The location of NF-*κ*B is within the cytosol in healthy cells as inactive forms that is bond to I*κ*B*α*. However, cancer cells have active forms of NF-*κ*B due to I*κ*B*α* phosphorylation. The active form of NF-*κ*B can induce prosurvival gene expression including inhibitors of apoptosis and result in uncontrol cell growth [38]. Previous studies have shown the inhibition effect of CNP on NF-*κ*B in different cell lines [39, 40]. Thus, inhibiting the NF-*κ*B by the downregulation effect of CNP on ROS levels can be a promising approach for cancer treatment.

Recent studies showed both antioxidant and oxidant roles of CNP in different cells. In agreement with our findings, Patel et al. showed the inhibitory effect of CNP on ROS levels and suggested that CNP has a potential therapeutic effect on human monocytic leukemia cells [26]. However, some previous studies demonstrated the antitumoral effect of CNP with increasing or even unchanged ROS levels. Contrary to our results Lin et al. demonstrated the dose-dependent and time-dependent effect of CNP on human lung cancer cell lines by increasing the ROS level [27]. Also, Park et al., showed the cytotoxic effect of CNP on cultured human epithelial cells by increasing ROS levels and decreasing antioxidant levels which induce cell apoptosis [28]. However, in Xiao et al.'s study, although the CNP induces a cytotoxic effect on gastric cancer cells, the ROS level was unchanged after CNP treatment [29]. These discrepancies can be due to different doses of CNP and also different cell lines. Also, it has been indicated that cell pH is an important key factor for the oxidant or antioxidant role of CNPs [41].

Interestingly, as previously noted, ROS has a double-edged sword function. Both elevating and lowering oxidant level has been suggested as a treatment strategy for cancer cells [42, 43]. In cancer cells, increasing levels of ROS as a result of signaling cascades and metabolic reactions may induce cellular antioxidant upregulation to maintain redox homeostasis. Therefore, exogenous ROS-producing agents can induce cancer cell deaths by triggering the ROS level [33]. On the other hand, as elevated levels of ROS play an important role in carcinogenesis, upregulation of cell antioxidants can deplete the ROS level and consequently cause growth inhabitation and cancer cell death [33].

It is worth mentioning that the least concentration of CNP that was needed to change the oxidant and antioxidant level was totally higher in the CSC-LC spheres in comparison to the YM1 cell line. Also, the IC_50_ for CNP was higher in the CSC-LC spheres in comparison to the YM1 cell line (968 and 758 *μ*M, respectively). These results can be explained by the potential treatment resistance features of CSC cells.

In summary, our study suggests the potential role of CNP as an effective anticancer treatment for EC and cancer stem cells. However, our study had some limitations. First, the signaling pathways of NF-*κ*B were not investigated. Second, the study was an *in vitro* examination, and further *in vivo* studies are missing. Further *in vivo* and clinical studies are recommended to highlight the effect of CNP on EC and cancer stem cells.

## 5. Conclusion

In conclusion, the present study showed that cerium oxide nanoparticles have potential anticancer effects on esophageal and cancer stem cells by increasing the cell antioxidant levels (including SOD, CAT, thiol, and TAC) and decreasing the oxidant levels (including ROS and MDA) in YM1 and CSC-LC spheres.

## Data Availability

The authors of this article will share all the data underlying the findings of their manuscripts with other researchers. This sharing of data allows researchers to replicate the results of an article and conduct secondary analyses. Therefore, I hereby declare the statement of “availability” for the data used in this manuscript. All the results were analyzed triplicate in comparison to the untreated control group. A *p* value lower than 0.05 was used for a statistically significant level. And researchers can communicate with the first author and the corresponding authors for the data by email.
